# Breakdown of Phosphatidylserine Asymmetry Following Treatment of Erythrocytes with Lumefantrine

**DOI:** 10.3390/toxins6020650

**Published:** 2014-02-20

**Authors:** Kousi Alzoubi, Bassel Alktifan, Gergely Oswald, Myriam Fezai, Majed Abed, Florian Lang

**Affiliations:** Department of Physiology, University of Tuebingen, Gmelinstr. 5, D-72076 Tübingen, Germany; E-Mails: kossai.z@gmail.com (K.A.); samosamosamo@hotmail.de (B.A.); gregor.oswald@gmx.de (G.O.); Myriam.fezai@gmail.com (M.F.); dr.magd81@hotmail.com (M.A.)

**Keywords:** phosphatidylserine, lumefantrine, calcium, ceramide, cell volume, eryptosis

## Abstract

Background: Lumefantrine, a commonly used antimalarial drug, inhibits hemozoin formation in parasites. Several other antimalarial substances counteract parasitemia by triggering suicidal death or eryptosis of infected erythrocytes. Eryptosis is characterized by cell shrinkage and cell membrane scrambling leading to phosphatidylserine-exposure at the erythrocyte surface. Signaling involved in eryptosis include increase of cytosolic Ca^2+^-activity ([Ca^2+^]_i_), formation of ceramide, oxidative stress and/or activation of p38 kinase, protein kinase C (PKC), or caspases. The present study explored, whether lumefantrine stimulates eryptosis. Methods: Cell volume has been estimated from forward scatter, phosphatidylserine-exposure from annexin V binding, [Ca^2+^]_i_ from Fluo3-fluorescence, reactive oxygen species from 2',7'-dichlorodihydrofluorescein-diacetate fluorescence, content of reduced glutathione (GSH) from mercury orange fluorescence, and ceramide abundance from binding of fluorescent antibodies in flow cytometry. Results: A 48 h exposure to lumefantrine (3 µg/mL) was followed by a significant increase of annexin-V-binding without significantly altering forward scatter, [Ca^2+^]_i_, ROS formation, reduced GSH, or ceramide abundance. The annexin-V-binding following lumefantrine treatment was not significantly modified by p38 kinase inhibitors SB203580 (2 μM) and p38 Inh III (1 μM), PKC inhibitor staurosporine (1 µM) or pancaspase inhibitor zVAD (1 or 10 µM). Conclusions: Lumefantrine triggers cell membrane scrambling, an effect independent from entry of extracellular Ca^2+^, ceramide formation, ROS formation, glutathione content, p38 kinase, PKC or caspases.

## 1. Introduction

Lumefantrine is one of the most commonly used drugs for the treatment of infections with *Plasmodium* [1,2,3]. Lumefantrine is at least in part effective by inhibiting hemozoin formation in the parasite [4]. At least in theory, lumefantrine may be effective in addition by triggering suicidal death of infected erythrocytes [5]. Following invasion of the erythrocyte, the pathogen activates several channels including Ca^2+^-permeable erythrocyte cation channels [6,7]. Activation of the channels is required for uptake of nutrients, Na^+^ and Ca^2+^, as well as for disposal of waste products [7]. Accordingly, intraerythrocyte survival of the pathogen depends on the activity of those channels [6,7]. The Ca^2+^ entry following activation of the Ca^2+^-permeable cation channels stimulates, however, the suicidal death of erythrocytes or eryptosis [5]. As eryptotic erythrocytes are rapidly cleared from circulating blood [8], the Ca^2+^ entry and subsequent eryptosis limits the life span of infected erythrocytes and, thus, counteracts the development of parasitemia [5]. Along those lines, eryptosis is accelerated in infected erythrocytes with sickle-cell trait, beta-thalassemia-trait, homozygous Hb-C, and G6PD-deficiency [9,10,11,12,13,14,15,16], genetic disorders known to confer partial resistance to malaria [10,11,17]. Similarly, eryptosis may be stimulated and the clinical course of malaria favorably influenced by iron deficiency [18], lead [19], chlorpromazine [20], and inhibition of NO synthase [21]. 

The hallmark of eryptosis is cell membrane scrambling [22], which may be triggered by increase of cytosolic Ca^2+^ concentration ([Ca^2+^]_i_). An increase of [Ca^2+^]_i_ may further lead to cell shrinkage due to activation of Ca^2+^-sensitive K^+^ channels with subsequent K^+^ exit, hyperpolarization, Cl^−^ exit, and, thus, cellular loss of KCl with osmotically obliged water [23]. Signaling of eryptosis further includes ceramide [22], caspases [24,25,26,27,28], and several kinases, including AMP activated kinase AMPK [29], casein kinase 1α [30,31], cGMP-dependent protein kinase [32], Janus-activated kinase JAK3 [33], protein kinase C [34], p38 kinase [35], PAK2 kinase [36], as well as sorafenib [37] and sunitinib [38] sensitive kinases.

The present study explored, whether lumefantrine influences [Ca^2+^]_i_, cell volume and cell membrane scrambling with phosphatidylserine exposure at the erythrocyte surface. The observations reveal that lumefantrine stimulates erythrocyte cell membrane scrambling, an effect at least in part due to increase of [Ca^2+^]_i_. 

## 2. Results and Discussion

**Figure 1:**
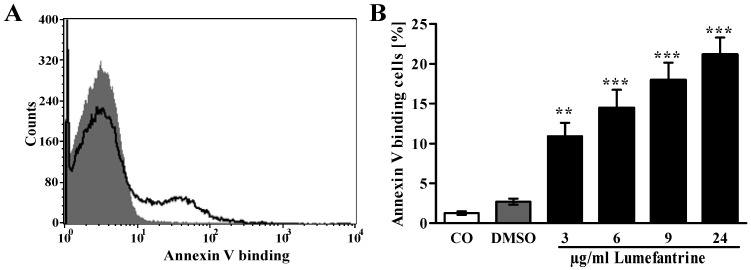
Effect of lumefantrine on phosphatidylserine exposure. (**A**) Original histogram of annexin V binding of erythrocytes following exposure for 48 h to Ringer solution without (grey shadow) and with (black line) presence of 24 µg/mL lumefantrine; (**B**) Arithmetic means ± SEM (*n* = 8) of erythrocyte annexin-V-binding following incubation for 48 h to Ringer solution without (white bar) or with (black bars) presence of lumefantrine (3–24 µg/mL) or, for comparison, DMSO (0.3%) alone (grey bar). ** (*p* < 0.01), *** (*p* < 0.001) indicate significant differences from the absence of lumefantrine (ANOVA).

The present study explored whether suicidal erythrocyte death or eryptosis is modified by lumefantrine. Eryptosis is characterized by breakdown of phosphatidylserine asymmetry of the erythrocyte cell membrane with cell membrane scrambling and subsequent increase of phosphatidylserine abundance at the cell surface. Thus, phosphatidylserine exposing erythrocytes were identified by annexin-V-binding in FACS analysis. As shown in Figure 1, a 48 h exposure to lumefantrine (≥3 µg/mL) significantly increased the percentage of annexin-V-binding erythrocytes. 

**Figure 2:**
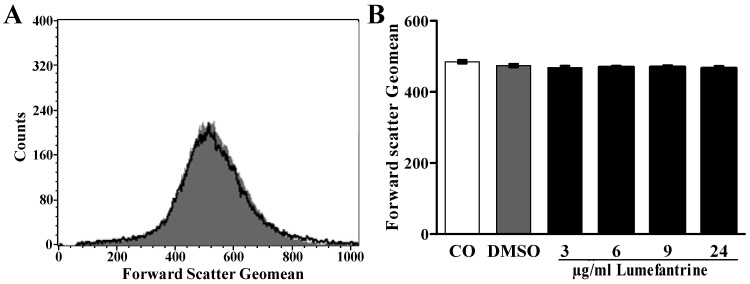
Effect of lumefantrine on erythrocyte forward scatter. (**A**) Original histogram of forward scatter of erythrocytes following exposure for 48 h to Ringer solution without (grey shadow) and with (black line) presence of 24 µg/mL lumefantrine; (**B**) Arithmetic means ± SEM (*n* = 8) of the normalized erythrocyte forward scatter (FSC) following incubation for 48 h to Ringer solution without (white bar) or with (black bars) lumefantrine (3–24 µg/mL) or, for comparison, DMSO (0.3%) alone (grey bar).

Eryptosis is further typically paralleled by cell shrinkage. Accordingly, cell volume was estimated from forward scatter in flow cytometry. As illustrated in Figure 2, a 48 h exposure to lumefantrine did not significantly modify erythrocyte forward scatter even at the highest concentrations (24 µg/mL) employed. 

**Figure 3:**
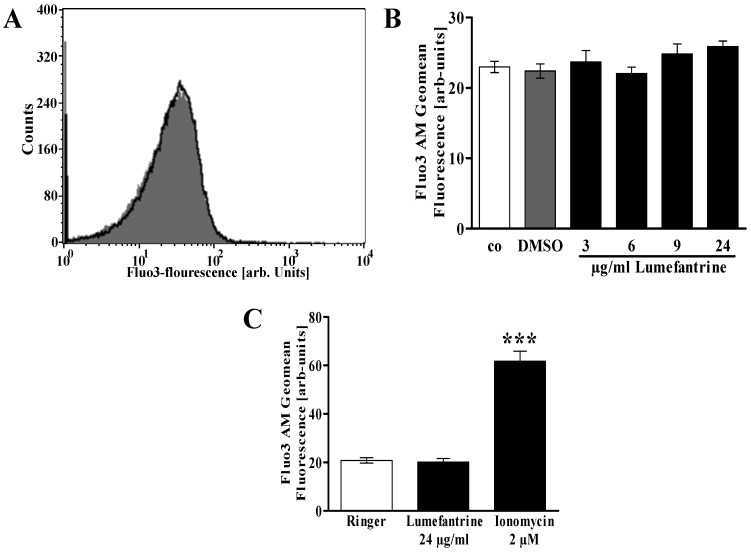
Effect of lumefantrine on erythrocyte cytosolic Ca^2+^ concentration. (**A**) Original histogram of Fluo3 fluorescence in erythrocytes following exposure for 48 h to Ringer solution without (grey shadow) and with (black line) presence of 24 µg/mL lumefantrine; (**B**) Arithmetic means ± SEM (*n* = 8) of the Fluo3 fluorescence (arbitrary units) in erythrocytes exposed for 48 h to Ringer solution without (white bar) or with (black bars) lumefantrine (3–24 µg/mL) or, for comparison, DMSO (0.3%) alone (grey bar); (**C**) Arithmetic means ± SEM (*n* = 4) of the Fluo3 fluorescence (arbitrary units) in erythrocytes exposed for 48 h to Ringer solution without (white bar) or with 24 µg/mL lumefantrine (black bar) or, for comparison, 2 µM ionomycin (grey bar). *** (*p* < 0.001) indicates significant difference from the absence of ionomycin (ANOVA).

Further experiments were performed to shed some light on the signaling involved in lumefantrine induced cell membrane scrambling. As cell membrane scrambling is stimulated by increase of cytosolic Ca^2+^ activity ([Ca^2+^]_i_), erythrocytes were loaded with Fluo3-AM and [Ca^2+^]_i_ estimated from Fluo3 fluorescence in FACS analysis. As shown in Figure 3, a 48 h exposure of human erythrocytes to lumefantrine did not appreciably modify Fluo3 fluorescence even at the highest concentrations (24 µg/mL) employed. 

As cell membrane scrambling could be triggered without increase of [Ca^2+^]_i_ by ceramide, additional experiments were performed to test whether lumefantrine increases ceramide formation. The abundance of ceramide at the erythrocyte surface was determined utilizing an anti-ceramide antibody. As a result, the ceramide abundance was similar following incubation of erythrocytes for 48 h without (11.40 ± 1.26 arbitrary units, *n* = 8) and with (11.87 ± 1.36 arbitrary units, *n* = 8) 9 µg/mL lumefantrine.

In order to test whether lumefantrine enhances oxidative stress, reactive oxygen species (ROS) were determined utilizing 2',7'-dichlorodihydrofluorescein diacetate (DCFDA), As a result, the DCFDA fluorescence was similar following incubation of erythrocytes for 48 h without (10.31 ± 0.60 arbitrary units, *n* = 4) and with (10.28 ± 0.53 arbitrary units, *n* = 4) 9 µg/mL lumefantrine. Oxidative stress is expected to decrease the abundance of glutathione (GSH). Thus, mercury orange was used for flow cytometric GSH measurement. As a result, the GSH level was similar in erythrocytes incubated for 48 h without (102 ± 8 arbitrary units, *n* = 5) and with (124 ± 9 arbitrary units, *n* = 5) 9 µg/mL lumefantrine, but was significantly (*p* < 0.001) decreased following exposure to oxidative stress by incubation of erythrocytes for 1 h with 200 µM t-Butylhydroperoxide (33 ± 4 arbitrary units, *n* = 5).

**Figure 4:**
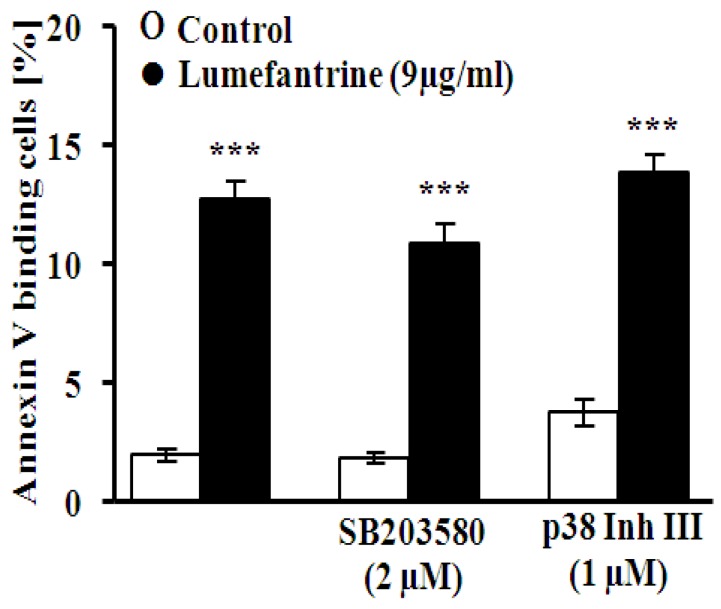
Effect of p38 kinase inhibitors on lumefantrine- induced annexin-V-binding. Arithmetic means ± SEM (*n* = 8) of the percentage of annexin-V-binding erythrocytes after a 48 h treatment with Ringer solution without (white bar) or with (black bars) 9 µg/mL lumefantrine in the absence (left bars) and presence of the p38 kinase inhibitors SB203580 (SB, 2 μM, middle bars) and p38 Inh III (Inh, 1 μM, right bars). *** (*p* < 0.001) indicates significant difference from the absence of lumefantrine (ANOVA).

An additional series of experiments was performed in order to explore whether the effect of lumefantrine on cell membrane scrambling required activation of p38 kinase. To this end, erythrocytes were exposed to 9 µg/mL lumefantrine for 48 h, either in the absence or presence of the p38 kinase inhibitors SB203580 (SB, 2 μM) and p38 Inh III (Inh, 1 μM). As illustrated in Figure 4, neither SB203580 nor p38 Inh III significantly modified the effect of lumefantrine on annexin V binding. 

**Figure 5:**
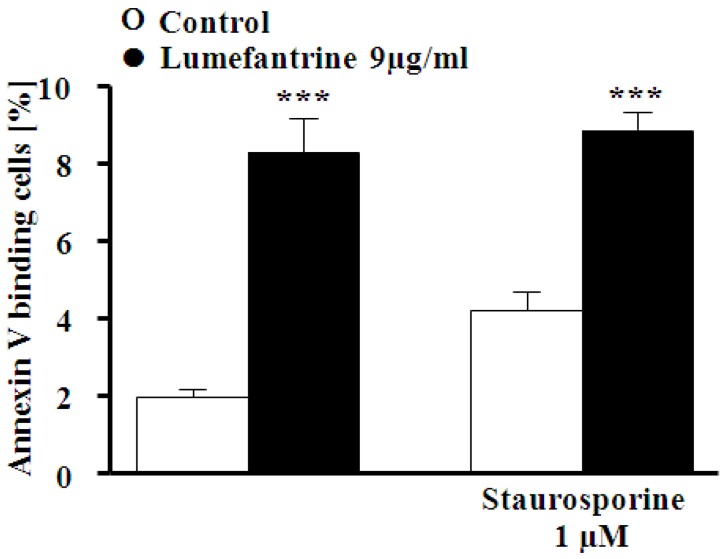
Effect of staurosporine on lumefantrine- induced annexin-V-binding. Arithmetic means ± SEM (*n* = 4) of the percentage of annexin-V-binding erythrocytes after a 48 h treatment with Ringer solution without (white bars) or with (black bars) 9 µg/mL lumefantrine in the absence (left bars) and presence (right bars) of PKC inhibitor staurosporine (1 μM). *** (*p* < 0.001) indicates significant difference from the absence of lumefantrine (ANOVA).

In order to test for an involvement of protein kinase C (PKC), erythrocytes were exposed to 9 µg/mL lumefantrine for 48 h either in the absence or presence of the unspecific PKC inhibitor staurosporine (1 μM). As illustrated in Figure 5, staurosporine did not significantly modify the effect of lumefantrine on annexin V binding. 

**Figure 6:**
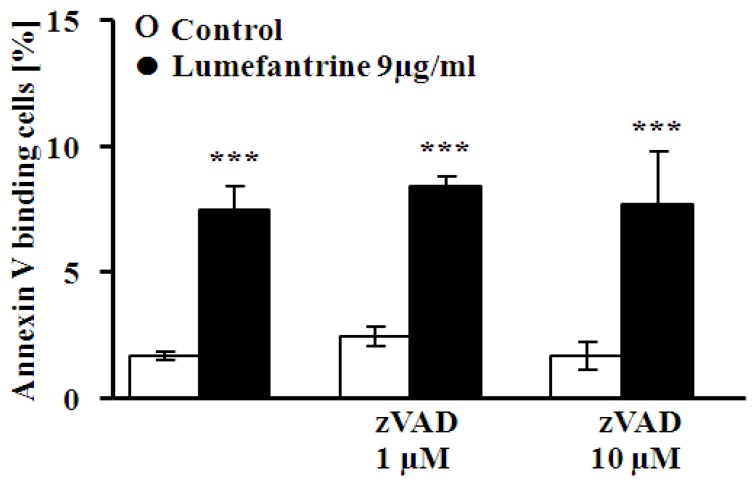
Effect of pancaspase inhibitor zVAD on lumefantrine- induced annexin-V-binding. Arithmetic means ± SEM (*n* = 4) of the percentage of annexin-V-binding erythrocytes after a 48 h treatment with Ringer solution without (white bar) or with (black bars) 9 µg/mL lumefantrine in the absence (left bars) and presence (middle and right bars) of the pancaspase inbitor zVAD (zVAD, 1 or 10 μM). *** (*p* < 0.001) indicates significant difference from the absence of lumefantrine (ANOVA).

A further series of experiments addressed the putative involvement of caspases. To this end, erythrocytes were exposed to 9 µg/mL lumefantrine for 48 h either in the absence or presence of the pancaspase inhibitor zVAD (1 or 10 μM). As illustrated in Figure 6, zVAD did not significantly modify the effect of lumefantrine on annexin V binding. 

The present study discloses a novel effect of lumefantrine, *i.e.*, the stimulation of cell membrane scrambling leading to phosphatidylserine translocation to the erythrocyte surface. The concentration required to significantly enhance the percentage of phosphatidylserine exposing erythrocytes is only slightly higher than that (2 µg/mL) observed *in vivo* [3].

Lumefantrine was effective without increasing cytosolic Ca^2+^ activity ([Ca^2+^]_i_). Accordingly, lumefantrine did not significantly modify erythrocyte forward scatter. Other triggers of eryptosis activate Ca^2+^ sensitive K^+^ channels [22] with subsequent K^+^ exit, cell membrane hyperpolarization, Cl^−^ exit and, thus, cellular loss of KCl with osmotically obliged water [23]. The effects counteract cell swelling and subsequent rupture of the cell membrane with release of cellular hemoglobin, which could be filtered in renal glomerula and subsequently occlude renal tubules [39].

Lumefatrine induced cell membrane scrambling was further not paralleled by increase of ceramide or ROS formation and did not require SB203580/p38 Inh III sensitive p38 kinase or staurosporine sensitive PKC. Moreover, the effect of lumefatrine on cell membrane scrambling did not require zVAD sensitive caspases. Caspases participate in the triggering of cell membrane scrambling by ROS but not by increase of [Ca^2+^]_i_ [22]. 

Whatever cellular mechanism involved in the triggering of cell membrane scrambling by lumefatrine, phosphatidylserine exposing erythrocytes bind to respective receptors of phagocytes, which engulf and degrade the defective erythrocytes [22]. As a result, phosphatidylserine exposing erythrocytes are rapidly cleared from circulating blood [22]. The clearance of phosphatidylserine exposing erythrocytes from circulating blood results in anemia, if enhanced formation of new erythrocytes does not match the accelerated loss of phosphatidylserine exposing erythrocytes [22]. 

Phosphatidylserine exposure further fosters the adherence of the affected erythrocytes to endothelial CXCL16/SR-PSO [40]. The adherence of phosphatidylserine exposing erythrocytes to the vascular wall compromises microcirculation [40,41,42,43,44,45]. In addition, phosphatidylserine exposing erythrocytes stimulate blood clotting and, thus, thrombosis [41,46,47].

The stimulating effect of lumefantrine on eryptosis could thus lead to the development of anemia and the triggering of thrombosis. On the other hand, the stimulation of eryptosis could contribute to the known [1,2,3] antimalarial effect of lumefantrine. Eryptosis of infected erythrocytes foster the engulfment by phagocytes with subsequent degradation not only of the erythrocyte but, as well, of the pathogen [5]. 

Similar to the effect of lumefantrine, a wide variety of xenobiotics stimulate eryptosis [22,38,48,49,50,51,52,53,54,55,56,57,58,59,60,61,62,63,64,65,66,67,68,69,70,71,72,73,74,75]. Moreover, enhanced eryptosis is observed in a variety of clinical conditions [22], such as diabetes [28,76,77], renal insufficiency [78], hemolytic uremic syndrome [79], sepsis [80], malaria [5], sickle cell disease [81], Wilson’s disease [82], iron deficiency [83], malignancy [84], phosphate depletion [85], and metabolic syndrome [68]. At least in theory, the parallel intake of the respective xenobiotics and the respective diseases may enhance the sensitivity of erythrocytes to the effects of lumefantrine.

## 3. Experimental Section

### 3.1. Erythrocytes, Solutions, and Chemicals

Fresh Li-Heparin-anticoagulated blood samples were kindly provided by the blood bank of the University of Tübingen. The study is approved by the ethics committee of the University of Tübingen (184/2003 V). The blood was centrifuged at 125 g for 20 min at 23 °C and the platelets and leukocytes-containing supernatant was disposed. Erythrocytes were washed in Ringer solution containing (in mM) 125 NaCl, 5 KCl, 1 MgSO_4_, 32 N-2-hydroxyethylpiperazine-N-2-ethanesulfonic acid (HEPES), 5 glucose, 1 CaCl_2_; pH 7.4. For the experiments erythrocytes were incubated *in vitro* at a hematocrit of 0.4% at 37 °C for 48 h. Where indicated, erythrocytes were exposed to lumefantrine (Sigma-Aldrich, Hamburg, Germany) at the indicated concentrations. In Ca^2+^-free Ringer solution, 1 mM CaCl_2_ was substituted by 1 mM glycol-bis(2-aminoethylether)-*N*,*N*,*N*',*N*'-tetraacetic acid (EGTA). 

### 3.2. FACS Analysis of Annexin-V-Binding and Forward Scatter

After incubation under the respective experimental condition, 50 µL cell suspension was washed in Ringer solution containing 5 mM CaCl_2_ and then stained with Annexin-V-FITC (1:200 dilution; ImmunoTools, Friesoythe, Germany) in this solution at 37 °C for 20 min under protection from light. In the following, the forward scatter (FSC) of the cells was determined, and annexin-V fluorescence intensity was measured with an excitation wavelength of 488 nm and an emission wavelength of 530 nm on a FACS Calibur (BD, Heidelberg, Germany).

### 3.3. Measurement of Intracellular Ca^2+^

After incubation erythrocytes were washed in Ringer solution and then loaded with Fluo-3/AM (Biotium, Hayward, CA, USA) in Ringer solution containing 5 mM CaCl_2_ and 5 µM Fluo-3/AM. The cells were incubated at 37 °C for 30 min and washed twice in Ringer solution containing 5 mM CaCl_2_. The Fluo-3/AM-loaded erythrocytes were resuspended in 200 µL Ringer. Then, Ca^2+^-dependent fluorescence intensity was measured with an excitation wavelength of 488 nm and an emission wavelength of 530 nm on a FACS Calibur.

### 3.4. Determination of Oxidative Status

ROS production was determined utilizing 2',7'-dichlorodihydrofluorescein diacetate (DCFDA) [86]. Briefly, the cells were suspended in FACS buffer and the fluorescence was analyzed with flow cytometry (FACS-calibur from Becton Dickinson; Heidelberg, Germany). DCFDA fluorescence intensity was measured in FL-1 with an excitation wavelength of 488 nm and an emission wavelength of 530 nm. In an additional series of experiments the content of reduced glutathione was determined using mercury orange. To this end, cells were spun down, incubated in PBS containing 40 µM of mercury orange (sigma Aldrich, Germany) for 30 min, washed once, and resuspended in 200 µL PBS. The fluorescence intensity was measured with flow cytometry (FACS-calibur from Becton Dickinson; Heidelberg, Germany) at an excitation wavelength of 488 nm and an emission wavelength of 576 nm.

### 3.5. Determination of Ceramide Formation

For the determination of ceramide, a monoclonal antibody-based assay was used. After incubation, cells were stained for 1 h at 37 °C with 1 µg/mL anti ceramide antibody (clone MID 15B4, Alexis, Grünberg, Germany) in PBS containing 0.1% bovine serum albumin (BSA) at a dilution of 1:10. The samples were washed twice with PBS-BSA. Subsequently, the cells were stained for 30 min with polyclonal fluorescein isothiocyanate (FITC) conjugated goat anti-mouse IgG and IgM specific antibody (Pharmingen, Hamburg, Germany) diluted 1:50 in PBS-BSA. Unbound secondary antibody was removed by repeated washing with PBS-BSA. The samples were then analyzed by flow cytometric analysis with an excitation wavelength of 488 nm and an emission wavelength of 530 nm. 

### 3.6. Statistics

Data are expressed as arithmetic means ± SEM. As indicated in the figure legends, statistical analysis was made using ANOVA with Tukey’s test as post-test and *t* test as appropriate. n denotes the number of different erythrocyte specimens studied. As different erythrocyte specimens used in distinct experiments are differently susceptible to triggers of eryptosis, the same erythrocyte specimens have been used for control and experimental conditions.

## 4. Conclusions

Lumefantrine triggers cell membrane scrambling with phosphatidylserine translocation to the erythrocyte surface, an effect not requiring Ca^2+^ entry, ceramide formation or p38 kinase activation.
